# Genetic and clinical landscape of Duchenne muscular dystrophy in Guatemala: insights from a national study

**DOI:** 10.3389/fgene.2025.1595423

**Published:** 2025-10-17

**Authors:** M. Orozco, E. Kestler, G. Ramirez, G. Silva, J. Cabrera, S. De la Vega, A. Al Khleifat

**Affiliations:** ^**1**^ Neuromuscular and Rare Diseases Unit, Department of Neurology and Neurosciences, Hospital General “San Juan de Dios”, Guatemala City, Guatemala; ^2^ Biomedical Sciences PhD, School of Post Graduate Studies, Faculty of Medical Sciences, Universidad de San Carlos de Guatemala, Guatemala City, Guatemala; ^3^ Centre for Epidemiological Research in Sexual and Reproductive Health, Hospital General “San Juan de Dios”, Guatemala, Guatemala; ^4^ Department of Neurology and Neurosciences, Hospital General “San Juan de Dios”, Guatemala, Guatemala; ^5^ Genetics Unit, Hospital de las Obras Sociales del Hermano Pedro, Antigua Guatemala, Guatemala; ^6^ Genetics Unit, Roosevelt Hospital, Guatemala, Guatemala; ^7^ Rehabilitation Unit, Foundation for the Welfare of the Disabled (FUNDABIEM), Quetzaltenango, Guatemala; ^8^ Maurice Wohl Clinical Neuroscience Institute, King’s College London, London, United Kingdom

**Keywords:** dystrophin gene, neuromuscular disease, X-linked, molecular variants, diagnostic delay, genetic variability

## Abstract

Duchenne muscular dystrophy (DMD) is a severe X-linked disorder caused by mutations in the *DMD* gene, and it has a global prevalence of 3.6 per 100,000 people. Despite its well-documented genetic basis, no previous studies have characterized DMD in Guatemala. We analyzed 33 genetically confirmed cases to estimate the prevalence, describe the mutation spectrum, and assess the clinical features. The prevalence was 0.61 per 100,000 men younger than 30 years. Symptoms began before the age of 5 years in 85% of cases, yet 60% cases were diagnosed after the age of 6 years, highlighting significant diagnostic delays. Deletions were the most common mutation (55%), followed by point mutations (30%) and duplications (15%), with two novel variants being identified. Most deletions clustered in the exon 45–55 hotspot. Nearly half of the cases were eligible for exon-skipping therapies. These findings reveal genetic heterogeneity in the Guatemalan population, substantial delays in diagnosis, and the need for improved access to genetic testing, targeted treatments, and a national DMD registry.

## 1 Introduction

Duchenne muscular dystrophy (DMD) is a severe, progressive, and disabling neuromuscular disease; it is one of the most common muscular dystrophies (Matsuo et al., 2016). It is considered an inherited, recessive, X-linked (Xp21) myopathy caused by mutations in the DMD gene (Huamán-Dianderas et al., 2019). The reported worldwide incidence is 1:3 per 600–9,300 live births (Schreiber-Katz et al., 2014). The worldwide prevalence has been estimated at 3.6 per 100,000 population, but it varies by the geographic area (Ryder et al., 2017).

DMD predominantly affects males due to its X-linked recessive inheritance pattern. As males are hemizygous for the X chromosome, the presence of a single pathogenic variant in the *DMD* gene is sufficient to cause the disease. Affected males cannot transmit the mutated allele to their sons but will pass it to all daughters, who become obligate carriers due to the inheritance of one affected X chromosome (Huamán-Dianderas et al., 2019).

Although rare, DMD can also occur in females. This may result from skewed X-chromosome inactivation favoring the expression of the X chromosome carrying the pathogenic *DMD* allele, from balanced X-autosome translocations disrupting the gene, or from the presence of biallelic pathogenic variants (Lee et al., 2015).

Countries with a high economic development index (HEDI) tend to have more robust epidemiological data, reflecting better access to diagnostic technologies and disease surveillance systems. In contrast, countries with medium or low EDI often lack such data, highlighting significant limitations. Nowhere is this more evident than in Guatemala, where no prevalence data for DMD are available. By comparison, Chile, a country with more developed healthcare infrastructure, reports a prevalence of 11.51 per 100,000 men younger than 30 years, illustrating the regional variation in both disease burden and the ability to measure it, as well as the detection capacity (Castiglioni et al., 2018; Salari et al., 2022). Another reported and related factor is the economic burden of this disease as it progresses to the critical stages of the disease, with the cost of the disease increasing by up to 16 times (Schreiber-Katz et al., 2014).

The molecular basis of DMD is complex, and over 7,000 variants in the DMD gene are thought to be associated with DMD, with deletions and duplications of exons being the most frequent variants at 80% and 20%, respectively, with point gene variants (Bladen et al., 2015). The DMD gene is 2.4 Mb in size and harbors 79 exons. This gene produces the protein dystrophin, which is part of the dystrophin-associated protein complex with a structural function in helping to stabilize the sarcolemma and protecting muscle fibers from damage and necrosis caused by prolonged contraction. When reduced but functional dystrophin is present, it is considered Becker muscular dystrophy (BMD-OMIM #300376), which carries a better prognosis for the patient (Iannaccone and Castro, 2013).

The structural and functional variations of dystrophin depend on the type of mutation in the DMD gene, which can range from an affected but functional protein to a nonfunctional or even absent protein. As a result, they can lead to different forms and severities of the disease. Identifying the mutational spectrum of the DMD gene is important to guide and apply feasible therapies that are mutation-specific. There is a lack of knowledge about the mutation profile in Guatemalan patients. Identifying the spectrum in the Guatemalan population is necessary to determine if they are eligible for treatment (Dwianingsih et al., 2023; Bidinost et al., 2018; Gao and McNally, 2015).

Currently, Guatemala lacks an epidemiological surveillance system for neuromuscular and rare diseases, and the genetic profile of patients affected by DMD is unknown. Many cases, especially in rural areas, are underdiagnosed. In this article, we aim to present the national urban–rural results of an epidemiological, sociodemographic, and clinical characteristics, and mutational spectrum in the Guatemalan population.

Understanding the genetic diversity of DMD is crucial for several reasons. It provides insights into genetic risk factors, offering valuable knowledge about previously unknown aspects of the disorder. Moreover, studying DMD in diverse populations helps to develop more inclusive and precise genetic tests, diagnostics, and therapies. Without expanding genetic research beyond Western populations, there is a significant risk of overlooking variants that are critical for understanding DMD in other ethnic and regional groups.

Currently, there is limited knowledge of the genetic profile of DMD in Guatemala and other Central American populations. Most genetic studies of DMD focus on regions with robust healthcare infrastructure, leaving gaps in our understanding of how the disease manifests in lower-resource settings. In this study, we seek to address these gaps by characterizing the genetic and clinical features of DMD in Guatemala, a population with a distinct socioeconomic and genetic background. By analyzing the prevalence, mutational spectrum, and clinical presentation of DMD in this region, we aim to identify novel genetic variants and assess whether previously known mutations are similarly implicated. Understanding these region-specific genetic factors could provide new insights into the disease mechanism and support the development of targeted therapies and diagnostic tools, thus contributing to a more comprehensive global understanding of DMD.

## 2 Methods

### 2.1 Data collection

During 16 months (October 2022 to January 2024), 33 (100%) active cases of any age with clinical suspicion of DMD were detected from two national reference hospitals (73%) that are the most important in the country, from the Social Security hospital (9%) and the Foundation for the Welfare of the Disabled (FUNDABIEM) (15%), and from private clinics (3%). Cases of suspected DMD without confirmed pathogenic mutations in the *DMD* gene were excluded. A follow-up, cross-sectional, non-probability, convenience-based, analytical study was performed.

For variant detection, multiplex ligation-dependent probe amplification (MLPA) was performed by probe denaturation, ligation, polymerase chain reaction (PCR) amplification, and fragment separation by capillary electrophoresis, using MRC Holland probes P034 and P035 that analyze all exons (79 exons), for deletions or duplications of the DMD gene. In cases where MLPA did not detect any deletions or duplications, sequencing of the gene was performed. Coffalyser.net software application was used for bioinformatic analysis of the data obtained (Coffa and den Berg, 2011). The DNB-SEQ400 next-generation mass sequencer was used for DNA sequencing analysis. The analysis was aimed at identifying variants included in the exonic regions or splice regions, insertions, and small deletions. Genetic testing was performed by the GenCell Genética Avanzada laboratory in Bogotá, Colombia, along with the logistics of transporting blood (79%) and saliva (21%) samples. A negative molecular test refers to the absence of detectable pathogenic variants in the *DMD* gene using standard diagnostic methods such as MLPA and NGS. Given the lack of prior data on the *DMD* gene mutational spectrum in Guatemala, no predefined variants were targeted; instead, all exons were analyzed using MLPA for large deletions/duplications and NGS for point mutations, splice-site variants, and small indels. Deep intronic regions were not assessed.

### 2.2 Data management and analysis

A data collection form that included demographic, social, economic, clinical, electrodiagnostic, and molecular laboratory studies was developed. All data were recorded and re-recorded for recording errors or inconsistencies. This was carried out in the statistical program EPINFO V6.0 (Camp et al., 2018), and descriptive statistics were obtained, with a confidence interval of 95%. The analysis and interpretation of genetic variants were performed using the open-access, web-based tool “DMD Open-access Variant Explore (DOVE)” (Bailey and Miller, 2019), providing information on amino acid change, functional predictions at the protein level, length of the mutated sequence, and molecular eligibility for treatment based on specific variants to correct the reading frame.

Diagnostic delay was defined as the time elapsed between the onset of the initial symptoms and the confirmation of diagnosis through genetic testing. Age distribution normality was assessed using the Shapiro–Wilk test.

### 2.3 Whole-genome sequencing

DNA was isolated from venous blood using standard methods. DNA concentration was set at 100 ng/ul, as measured by a fluorimeter. DNA integrity was assessed using gel electrophoresis. All samples were sequenced using Illumina’s FastTrack services (San Diego, CA, United States) on the Illumina HiSeq 2000 (100 bp paired-end reads) and HiSeqX platforms (150 bp paired-end reads) using PCR-free library preparations. Binary sequence alignment/map formats (BAM) were generated for each individual. The Project MinE genomes were aligned with Isaac (Illumina) to hg19.

### 2.4 Quality control

Sample mismatch was tested using sex checks, where genetic sex was compared to the reported gender. After quality control, the full set of gVCFs (genomic variant call formats) was merged together by first converting the gVCFs into the Plink format and then merging all files together. This generated a single dataset containing all variant sites across all individuals. Non-autosomal chromosome and multi-allelic variants were excluded from pilot analyses.

## 3 Results

Thirty-seven cases with clinically suspected DMD were detected and referred from different health institutions participating in the study. Four cases had negative genetic test results and were therefore excluded. This left 33 genetically confirmed cases with DMD that are part of this analysis. Again, it is confirmed that all genetically positive cases were male as DMD is an X-linked disease.

In this cohort, the estimated prevalence of genetically confirmed DMD was 0.61 per 100,000 males younger than 30 years (33 cases among 5,395,769 males, based on 2018 Guatemalan census projections) (Guatemala. Instituto Nacional de Estadística, 2019). Clinical data (Table 1) indicated an age range of 3–25 years, with a mean age of 9 years. Delayed motor milestones were observed in 57.5% of cases, and 85% experienced symptom onset before the age of 5 years, most commonly presenting with muscle weakness and a positive Gowers’ sign. At the time of evaluation, only 33.3% had lost ambulation.

**TABLE 1:** Clinical characteristics found in patients with DMD in Guatemala.

No.	Sex	Age	Age of onset	Diagnosis age	Diagnosis delay	Motor milestone delay	Consultation symptom
	M or F	years	years	years	(i.e., time from onset of symptoms to diagnosis in months)	(i.e., yes or no)	(first symptoms that parents noticed)
1	M	8	3	6	36	Yes	Frequent drops
2	M	6	4	6	24	No	Muscle weakness and frequent falls
3	M	10	3	9	72	Yes	Muscle weakness and frequent falls
4	M	13	4	13	108	Yes	Tiptoeing
5	M	10	6	10	48	No	Muscle weakness
6	M	7	2	6	48	Yes	Muscle weakness and frequent falls
7	M	9	4	9	60	Yes	Frequent drops
8	M	9	7	9	24	No	Tiptoeing
9	M	10	7	10	36	No	Muscle weakness
10	M	10	4	9	60	No	Tiptoeing
11	M	14	5	7	24	No	Tiptoeing
12	M	5	3	5	24	Yes	Muscle weakness
13	M	5	2	5	36	Yes	Muscle weakness and frequent falls
14	M	8	3	8	60	No	Tiptoeing
15	M	3	3	6 months	0	No	Tiptoeing
16	M	8	4	7	36	No	Tiptoeing and frequent falls
17	M	7	4	7	36	Yes	Muscle weakness
18	M	25	3	4	12	No	Frequent drops
19	M	7	4	6	24	No	Muscle weakness
20	M	9	4	7	36	Yes	Muscle weakness and frequent falls
21	M	7	2	4	24	Yes	Muscle weakness
22	M	6	3	4	12	No	Muscle weakness
23	M	12	4	7	36	Yes	Muscle weakness
24	M	10	2	9	84	Yes	Muscle weakness and frequent falls
25	M	7	2	7	60	Yes	Muscle weakness and frequent falls
26	M	7	4	7	36	No	Muscle weakness and frequent falls
27	M	10	2	10	96	Yes	Muscle weakness and frequent falls
28	M	12	2	2	0	Yes	Muscle weakness
29	M	5	2	5	36	Yes	Muscle weakness
30	M	8	2	4	24	Yes	Muscle weakness and frequent falls
31	M	8	3	8	60	Yes	Muscle weakness and frequent falls
32	M	9	2	6	48	Yes	Tiptoeing
33	M	10	3	9	72	No	Muscle weakness and frequent falls

Creatine phosphokinase (CPK) levels were elevated in all patients who were tested, ranging from 345 to 16,000 U/L (normal range 20–200 U/L). Elevated values of creatinine kinase (CPK), as well as alanine and aspartate transaminase (ALT and AST), result from the rupture and necrosis of muscle fibers. As they are used as biomarkers, they are a guideline for genetic testing (Chien et al., 2022).

The mean diagnostic delay, from symptom onset to confirmed diagnosis, was 42 months (95% IC 33.21, 51.15) and the median was 36 months; however, one case experienced a 108-month delay. Only two patients were diagnosed promptly at symptom onset or earlier due to a known family history of DMD. The remaining cases experienced substantial delays, with over 90% cases being diagnosed for more than 12 months after symptom onset. The Shapiro–Wilk test indicated that the data did not follow a normal distribution (p = 0.0017). Therefore, a nonparametric Wilcoxon signed-rank test was applied, which confirmed that the delay was statistically significant (p < 0.05, α = 0.05).

Sociodemographic analysis revealed that 55% of families had a medium economic status, 42% had a low status, and 3% had a high status. A majority of patients (61%) were from rural areas. Educational delays were notable: 48% were not at the educational level expected for their age and 27% were not enrolled in school. A history of consanguinity was reported in 15% of cases.

Table 2 presents the details of the molecular variants of the 33 cases detected. A total of 23 (70%) cases presented deletion and duplication detected by MLPA, with deletions of one or more exons^1^ (55%) being the most frequent pathogenic variant detected, and most of them (72%) were found in one of the hotspots from exons 45 to 55. Six cases had deletions of a single exon, four of which were in exon 45, one case in exon 50, and one case in exon 30, which was the only one found within the in-frame.

**TABLE 2:** Spectrum of mutations found in patients with DMD in Guatemala.

No.	Deleted exon^a^	Exon duplicated^b^	Exon with variant type^c^	Variant type^d^	DNA level change^e^	Amino acid change^f^	Length of mutated sequence^g^	Potential therapy^h^
1	45–50			Frameshift		p.Glu2147Leu*9		Exon 51 skip
2		2		Frameshift	c.32–2_93+?dup	p.Phe32Metfs*15	62	
3	46–55			Frameshift	c.6615-?_8217+?del	p.Leu2206Thrfs*24	1,603	Exon 45 skip
4			30	Frameshift	c.4099dup	p.Gln1367ProfsTer10	1	
5			3	Frameshift	c.6986dup	p.Leu2330AlafsTer10	1	
6			40	Frameshift	c.5704_5707del	p.Ser1902TyfsTer10	4	
7		8–16		Frameshift	c.650-?_1992+?dup	p. ?		
8	3–7			Frameshift	c.94-?_649+?del	p.Phe32Metfs*13	556	Exon 8 skip
9	45			Frameshift	c.6439-?_8390+?del	p.Glu2147Alafs*17	176	Exon 44 skip
10	45–56			Frameshift		p.Glu2147Valfs*8	1,952	Exon 44 skip
11	45			Frameshift	c.6439-?_8390+?del	p.Glu2147Alafs*17	176	Exon 44 skip
12			40	Nonsense	c.5641C>T	p.Gln1881Ter	1	Ataluren
13	49–50			Frameshift	c.7099-?_7309+del	p.Glu2367Leufs*9	211	Exon 51 skip
14			5	Nonsense	c.354G>A	p.Trp118Ter	1	Ataluren
15			5	Nonsense	c.354G>A	p.Trp118Ter	1	Ataluren
16	45			Frameshift	c.6439-?_8390+?del	p.Glu2147Alafs*17	176	
17		8–12		Frameshift		p. ?		
18			5	Nonsense	c.354G>A	p.Trp118Ter	1	Ataluren
19	46–51			Frameshift		p.Leu2206Glnfs*23	928	Exon 45 skip
20		2–7		Frameshift				
21	46–50			Frameshift	c.6615-?_7309+del	p.Arg2205Serfs*16	695	Exon 51 skip
22	5–7			Frameshift	c.265-?_649+?del	p.Val89Metfs*13	385	Exon 8 skip
23	10–11			Frameshift	c.961-?_1331+?del	p.His321Phefs*3	371	Exon 12 skip
24	45			Frameshift	c.6439-?_8390+?del	p.Glu2147Alafs*17	176	Exon 44 skip
25			?	Nonsense	c.7683G>A	p.Trp2561Ter	1	Ataluren
26	50			Frameshift		p.Arg2401Leufs*9	109	Exon 51 skip
27	48–50			Frameshift	c.(6912 + 1_6913–1)-(7309 + 1_1310–1)del	p.Val2305Leufs*9	397	Exon 51 skip
28		5–6		Frameshift				
29	49–52			Frameshift	c.7099-?_7660+?del	p.Glu2367Leufs*22	562	Exon 53 skip
30			6	Nonsense	c.488G>A	p.Trp163Ter	1	Ataluren
31	49–50			Frameshift		p.Glu2367Leufs*22	211	Exon 51 skip
32	30			In-frame	c.4072-?_4233+?del	p.Ala1357_Lys1411del	162	
33			34	Nonsense	c.4839G>A	p.Trp1613Ter	1	Ataluren

^a^Deleted exon: deletion or absence of one or more exons.

^b^Exon duplicated: repetition of one or more exons.

^c^Exon with point mutation: Number of variant exon type, this change can be deletion, duplication, insertion, missense, or nonsense.

^d^Variant type: A mutation or pathogenic variant frameshift, outframe or in‐frame.

^e^DNA level change: change in DNA sequence.

^f^Amino acid change: one amino acid (building block of proteins) is replaced by a different amino acid.

^g^Length of mutated sequence: number of nucleotides that have changed in a DNA sequence.

^h^Potential Therapy: treatment that shows the ability to be effective in modifiying a disease.

Of the total cases, 15% had a duplication of one or more exons, usually in the range between the first exon and exon 20. The largest duplication involved nine consecutive exons (exons 8–16), with each being duplicated once. The smallest was a single-exon duplication (exon 2), which was identified in one patient.

Next-generation sequencing revealed point mutations in 10 patients (30%). Of all cases, 21% were single-nucleotide variants that introduced premature stop codons (nonsense mutations), 76% were frameshift mutations, and 3% were in-frame mutations that did not alter the reading frame.

A single-exon deletion (exon 30) was detected and cataloged as an in-frame variant (NM_004006.2 c.4072-_4233+?del; p. Ala1357_Lys1411del). It presented a discrepancy between the genotype^2^ and the phenotype^3^, given that it was a case of a 9-year-old boy with a DMD phenotype and with a severe clinical condition due to the loss of gait at an early age. It would be expected that the pathogenic variant being in-frame would lead to a mild DMD phenotype (Zinina et al., 2022; Straub and Guglieri, 2023). However, this case is an exception to the in-frame rule.

Single-nucleotide variants, also known as point mutations (Gan et al., 2022), comprised 30% of all mutations in our cohort. These variants were evenly distributed in the gene and not specifically in hotspots. Notably, the most frequent of these types of mutations were nucleotide substitutions that generated premature stop codons (nonsense variants). Previous studies in individuals with point mutations were reported to be distributed along the *DMD* gene (Zimowski et al., 2021). However, it has been mentioned that in nonsense mutations, the position has important implications for predicting the phenotype. Therefore, at a certain position, it could cause mild phenotypes as mutations at the C-terminal end (exons 72–76) are not as detrimental as a truncated but partially functional protein is produced, showing a BMD phenotype (Torella et al., 2020).

Two frameshift variants that have not been previously reported in CLINVAR and LOVD were detected (Leiden Open Variation Database) (National Library of Medicine, 2025). One was a deletion from exon 3 to 7 (NM_004006.2 c.94-?_649+?del; p.Phe32Metfs*13) with the DMD phenotype, even with ambulation at 10 years of age. Another was a single-nucleotide variant in exon 30 that generates a stop codon (NM_004006.3 c.4099dup) and makes a change in the p.Gln1367ProfsTer10 protein, with a severe DMD phenotype and loss of ambulation at 9 years of age.

Another case of a single-nucleotide variant was in exon 3 (NM_004006.4 c.177del), which makes a p.Gln60LysfsTer15 protein change that is reported in the CLINVAR database but without functional evidence; therefore, it is probably pathogenic. In this case, it was a DMD phenotype with elevated muscle enzymes and even with ambulation at 11 years of age. These findings confirm that DMD presents genotypic heterogeneity and the possibility of discordance between genotype and phenotype in this disease. These new variants will be reported in the CLINVAR database as findings specific to the population studied.

## 4 Discussion

The findings of this study underscore significant diagnostic delays in the identification of DMD in Guatemala. Symptom onset occurred before the age of five years in 85% of cases, yet 60% were diagnosed after the age of six years. This reflects a substantial gap between disease onset and diagnosis, which is likely attributable to limited access to molecular diagnostics, low awareness of neuromuscular disorders among healthcare providers, and insufficient public health policies addressing rare diseases. As DMD is a progressive disorder, delayed diagnosis has critical implications and prevents timely intervention with supportive care and emerging disease-modifying treatments.

The study confirms that the mutational spectrum of Guatemalan DMD patients is consistent with global trends, with deletions (55%) being the most prevalent mutation type, primarily clustered within the exon 45–55 hotspot. However, the observed frequency of nonsense mutations (21%) is notably higher than the 10%–15% reported in other populations (Bladen et al., 2015; Braga et al., 2023). This finding is particularly relevant for the potential application of premature termination codon-readthrough therapies, such as ataluren, which could offer treatment options for a considerable proportion of patients (Mercuri et al., 2023; McDonald et al., 2022).

The presence of two novel pathogenic variants (Table 1; cases 4 and 8) in this cohort reinforces the importance of region-specific genetic research. While most deletions were in known hotspots, these novel variants highlight the genetic diversity in the Guatemalan population and the potential for previously unreported mutations. Such findings emphasize the need for population-wide genetic screening to capture the full spectrum of *DMD* gene alterations.

One of the key challenges identified is the lack of a national epidemiological surveillance system for DMD. Currently, there is no systematic registry of diagnosed cases, hindering effective disease monitoring and the development of national health strategies. Without a robust registry, estimating true disease prevalence and evaluating the long-term impact of therapeutic interventions remain difficult. Establishing a national DMD registry would facilitate patient follow-up, improve access to emerging treatments, and support clinical trial recruitment.

Furthermore, disparities in healthcare access, particularly between urban and rural populations, are highlighted in the study. More than 60% of patients were from rural areas, where healthcare resources are often limited. This geographical disparity contributes to later diagnoses and reduced access to genetic testing. Cost-effective diagnostic methods, such as MLPA for detecting exon deletions and duplications, could be widely implemented to bridge this gap. However, cases with point mutations or deep intronic variants require next-generation sequencing, which remains inaccessible to many Guatemalan patients due to financial and logistical constraints.

From a therapeutic perspective, 49% of cases in this study were eligible for exon-skipping therapies targeting exons 44, 45, 51, and 53, which are currently in clinical use or trials (Patterson et al., 2023; Fortunato et al., 2021; Takeda et al., 2021) The development of exon-skipping therapies represents a significant advancement in precision medicine, allowing mutation-specific treatment for a subset of DMD patients. Additionally, gene therapy approaches, such as micro-dystrophin replacement strategies and CRISPR-Cas9 genome editing, are in advanced clinical trials (Mendell et al., 2024; Happi et al., 2022). Expanding access to these therapies in Guatemala will require coordinated efforts at the governmental and international levels, including regulatory approvals and funding initiatives.

The study also reinforces the role of genetic counseling in patient management. Many affected families lack access to adequate genetic counseling, leading to misunderstandings about disease inheritance, recurrence risks, and reproductive options. Expanding genetic counseling services should be a priority to support the affected families and inform clinical decision-making.

## 5 Conclusion

In this study, we provide the first systematic genetic and clinical characterization of DMD in Guatemala. The findings confirm significant diagnostic delays and substantial genetic heterogeneity, including two novel frameshift variants: a multi-exon deletion (exons 3–7) and a single-nucleotide duplication in exon 30, associated with severe DMD phenotypes. A high proportion of nonsense mutations suggests that premature termination codon-readthrough therapies could benefit many patients. Nearly half of the cases were eligible for exon-skipping therapies, reinforcing the need for targeted treatments.

The absence of a national patient registry and limited access to genetic testing hinder timely diagnosis and treatment. Establishing a registry, improving diagnostic infrastructure, and increasing access to molecular therapies should be healthcare priorities. Genetic counseling services must also be expanded to better support the affected families.

Future research should focus on longitudinal follow-up, treatment responses, and the socioeconomic impact of DMD in Guatemala. Addressing these challenges will require coordinated efforts at the national and international levels to improve patient outcomes and healthcare accessibility.

## 6 Limitations

This study has several limitations. The sample size was relatively small (n = 33), limiting the generalizability of the findings to the broader Guatemalan population. Additionally, case ascertainment relied on a convenience-based sampling approach, potentially underestimating true disease prevalence due to unreported or undiagnosed cases, particularly in rural areas with limited healthcare access.

Genetic testing was restricted to multiplex ligation-dependent probe amplification and NGS, which may not detect all pathogenic variants, such as deep intronic mutations or structural rearrangements. The reliance on a single bioinformatics pipeline could have led to the omission of complex variants. Moreover, access to molecular testing was limited, making it likely that some cases remain undiagnosed.

This study was cross-sectional, providing a snapshot of disease prevalence and mutation distribution but lacking longitudinal data on disease progression, response to treatment, and long-term outcomes. Future studies should incorporate follow-up data to better assess clinical trajectories and the impact of therapeutic interventions.

Finally, the lack of a national DMD registry restricts epidemiological surveillance and complicates patient follow-up. Establishing a registry would enable more accurate prevalence estimates, facilitate clinical trial recruitment, and improve long-term patient management.

## Data Availability

The original contributions presented in this study are publicly available. These data can be found in the Leiden Open Variation Database 3 (LOVD3) repository, the access link is https://databases.lovd.nl/shared/individuals/00466679.

## Footnotes

1 Coding region of the gene

2 Genes encoding the physical characteristics of an organism

3 Characteristics observed as a result
